# A pharmacovigilance study of sirolimus-associated adverse events in organ transplantation

**DOI:** 10.3389/fimmu.2026.1780105

**Published:** 2026-06-03

**Authors:** Da-Zhuang Yi, Yan Jiao, Cai-Feng Xiu, Lu Han, Shi-Long Li, Xiao-Feng Sun, Bo Liu

**Affiliations:** 1 Department of Scientific Research, The First Hospital of Jilin University, Changchun, Jilin, China; 2 Department of Hepatobiliary and Pancreatic Surgery, General Surgery Center, The First Hospital of Jilin University, Changchun, Jilin, China; 3 Department of Cadre’s Wards Ultrasound Diagnostics, Ultrasound Diagnostic Center, The First Hospital of Jilin University, Changchun, Jilin, China; 4 Department of Orthopedic, Jilin Province Tumor Hospital, Changchun, Jilin, China

**Keywords:** adverse drug reactions, FAERS database, pharmacovigilance signals, rapamycin, sirolimus

## Abstract

**Background:**

Sirolimus (rapamycin), an antibiotic discovered in the 1970s, can effectively reduce allograft rejection, and plays an important role in the fields of immunosuppressive therapy and organ transplantation. Therefore, this study aimed to analyze adverse drug reactions (ADRs) associated with sirolimus using the FDA Adverse Event Reporting System (FAERS) database to inform clinical risk minimization strategies and guide safer therapeutic use.

**Methods:**

ADRs associated with sirolimus were retrospectively extracted from the FAERS database. Following the removal of duplicate entries, the final analytical dataset comprised 8,150,023 unique reports. Statistical analyses for signal detection of disproportionate reporting were performed using established pharmacovigilance metrics.

**Results:**

A total of 4,821 FAERS reports associated with sirolimus as the primary suspect drug were identified. Injury, poisoning and procedural complications (ROR = 1.61) \are the most common reports, followed by investigation (ROR = 2.19), infections and infestations (ROR = 1.74). The top ADRs matched known profiles, including kidney transplant rejection (ROR = 230.41). The analysis also revealed signals not prominently described in current prescribing information, including ovarian adenoma (ROR = 56.6), which should be interpreted cautiously as a hypothesis-generating pharmacovigilance signal rather than evidence of causality.

**Conclusion:**

This analysis details the ADR profile of sirolimus, revealing both known and potentially novel clinically significant adverse event signals that augment its known safety profile. Ongoing pharmacovigilance and targeted investigations are necessary to confirm these pharmacovigilance signals and inform optimal safety management strategies for sirolimus.

## 1 Introduction

Advances in surgical technology have established organ transplantation as the primary therapeutic intervention for end-stage organ failure, significantly enhancing patient quality of life (1–3). Despite considerable success, immune rejection remains a significant barrier to the long-term survival of transplant recipients (4, 5). Timely intervention is critical for maintaining graft function, with the administration of immunosuppressive agents (ISAs) being essential for preventing rejection (6, 7).

Sirolimus, also known as rapamycin, an antibiotic discovered in the 1970s from Streptomyces hygroscopicus on Easter Island (Rapanui), can effectively mitigate allograft rejection following organ transplantation by inhibiting mTOR (mechanistic target of rapamycin) signaling pathway to block the activation of T cells and B cells, which plays an important role in the fields of immunosuppressive therapy and organ transplantation, especially in renal transplantation (8, 9). Furthermore, recent research indicates that sirolimus plays a significant role in adjuvant treatment of tumors, prolonging the survival of cancer patients, preventing stent stenosis after coronary angioplasty and prolonging life (10–12).

Though the therapeutic benefits of sirolimus have been fully proved, its expanding clinical application needs to be highly vigilant about its safety. Although its efficacy, it is associated with recognized adverse drug reactions (ADRs), including gastrointestinal disorders, pulmonary toxicity, proteinuria, peripheral edema, and increased risk of rejection (13–15). However, the existing evidence describing these adverse events usually comes from studies which are lacking large-scale, real-world pharmacovigilance data. The FDA Adverse Event Reporting System (FAERS) database provides a powerful resource to solve these limitations.It facilitates systematic signal detection through quantitative disproportionality analysis, enabling the identification of potential associations between specific drugs (such as sirolimus) and specific ADRs with greater statistical power (16, 17).

Therefore, this study utilized the FAERS database to describe ADRs related to sirolimus, aiming to provide evidence-based insights for optimizing risk-benefit assessment, and to provide information for clinical and regulatory decision-making of sirolimus treatment.

## 2 Methods

### 2.1 Data source

This retrospective pharmacovigilance study utilized data from the FAERS database (https://open.fda.gov/data/). FAERS represents a comprehensive repository of spontaneous adverse event reports submitted to the FDA. Data extraction and deduplication were performed according to the FDA’s recommended methodology (18). The final analytical cohort comprised 8,150,023 unique reports spanning the period from 2004 to 2024. This extensive dataset provides a robust foundation for investigating ADRs associated with sirolimus.

### 2.2 Data extraction

A systematic extraction process that identified sirolimus-related ADRs was carried out, as described in our previous work (19). To ensure data integrity, duplicate reports were resolved using the FDA-recommended deduplication strategy: for each unique CASEID, the report with the most recent FDA_DT was retained; when CASEID and FDA_DT were identical, the record with the highest PRIMARYID was selected. Reports lacking essential demographic information were excluded to improve data quality. For disproportionality analysis, the background comparator included all deduplicated reports within the FAERS database, and reports listing sirolimus as the primary suspect (PS) drug constituted the exposure group.

The preliminary filtering produced 4,821 case reports that sirolimus was definitely used as PS drug. From these reports, 16,173 unique Preferred Terms (PTs) coded by terms in the Medical Dictionary of Regulatory Activities (MedDRA) were identified as related to sirolimus exposure. PTs provide standardized nomenclature for adverse events, which is essential for consistent pharmacovigilance analysis. Because a FAERS report may contain multiple PT, the report level count and PT level count are analyzed separately throughout the study to avoid ambiguity. The time to onset was determined by subtracting START_DT from EVENT_DT. Reports with missing, invalid, or contradictory dates were excluded from onset-time analyses.

### 2.3 Statistical analysis

**Figure 1:**
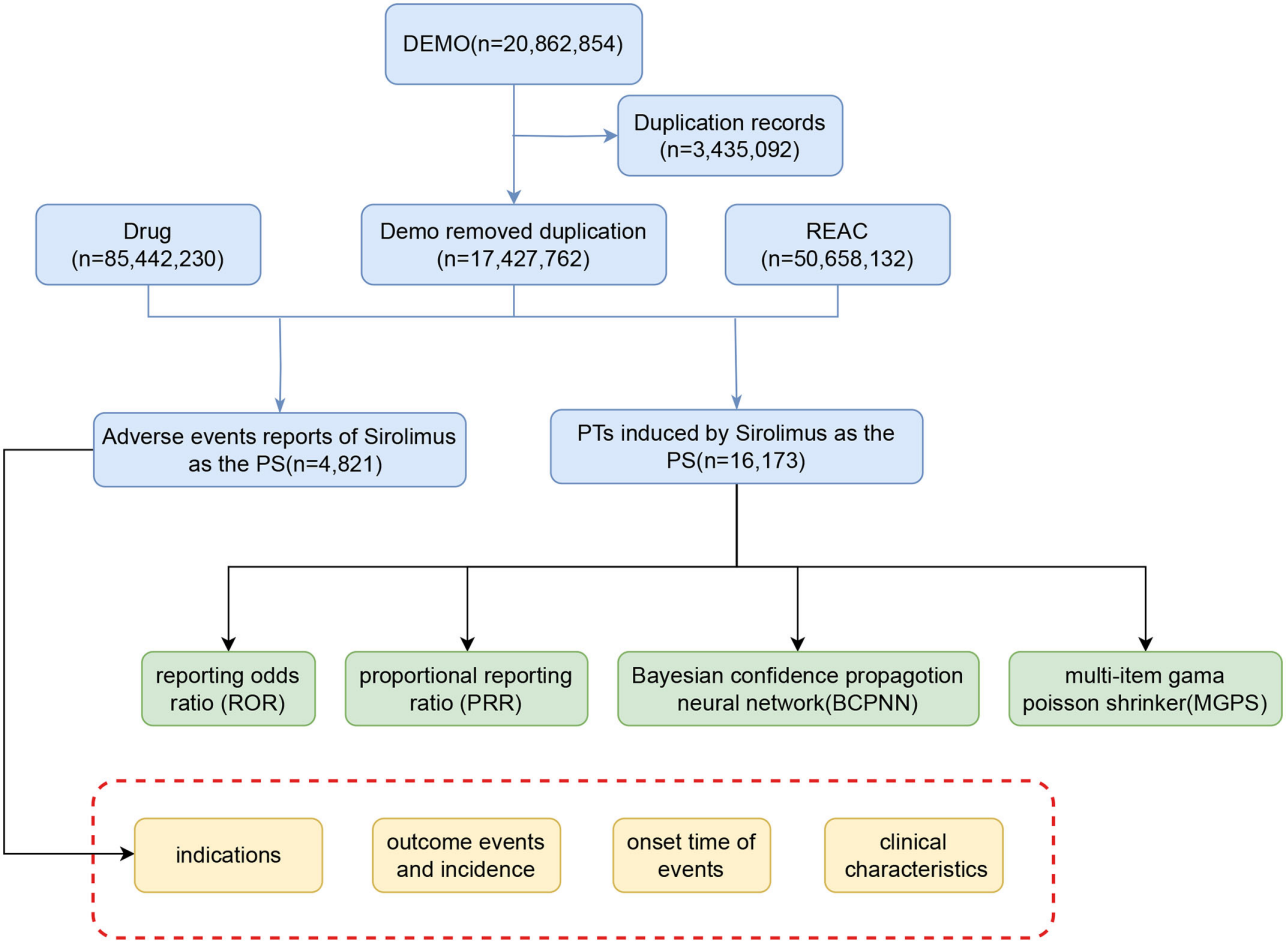
Flow diagram for the selection of sirolimus-related adverse events from FAERS.

Disproportionality analysis was performed using established pharmacovigilance methods to quantify signal strength and identify significant adverse event associations. The Reporting Odds Ratio (ROR) with 95% confidence intervals (CI) assessed association strength between sirolimus and specific adverse drug reactions (ADRs). The Proportional Reporting Ratio (PRR) compared sirolimus-related ADR frequencies against background reporting rates for all other drugs in the FAERS database. To enhance signal robustness, the Bayesian Confidence Propagation Neural Network (BCPNN) method was implemented, generating Information Components (IC) with Bayesian confidence intervals to evaluate dependency probabilities. Additionally, the Empirical Bayesian Geometric Mean (EBGM) quantified disproportionate reporting while adjusting for database-wide covariate patterns, with EBGM05 (lower 90% CI bound) used as the significance threshold. Signal detection criteria were defined as follows: ROR lower 95% confidence interval > 1; PRR ≥ 2 with χ² ≥ 4; IC025 > 0; and EBGM05 > 2. Signals meeting all four criteria were considered statistically significant disproportionate reporting signals. All statistical analyses were performed using R software (version 4.3.2). **Figure 1** illustrates the analytical workflow.

## 3 Results

### 3.1 General features on adverse drug reactions

**Table 1**; **Figure 2** summarize the general characteristics of patients reporting sirolimus-associated ADRs. A total of 4,821 FAERS reports associated with sirolimus as the primary suspect drug were documented, peaking in 2005. Most reports involved males (55.11%) and patients aged 18–60 years (40.03%). Geographically, the majority originated from the Americas (81.13%). The primary reporter source was consumers (29.72%). The most frequently reported outcome was other serious medical events (42.47%), followed by hospitalization (39.25%).

**Table 1:** Baseline characteristics of patients reporting adverse events related to Sirolimus from the FAERS database.

Variable	N (%)
Gender	
female	1802 (37.38%)
male	2657 (55.11%)
unknown	362 (7.51%)
Age	
<18 years	290 (6.02%)
18~60 years	1930 (40.03%)
>=60 years	1418 (29.41%)
unknow	1183 (24.54%)
Reporter	
Consumer	1433 (29.72%)
Physician	1274 (26.43%)
unknown	1100 (22.82%)
Other health-professional	639 (13.25%)
Pharmacist	369 (7.65%)
Lawyer	6 (0.12%)
Reported countries	
United States	2089 (81.13%)
Canada	124 (4.82%)
France	82 (3.18%)
other	74 (2.87%)
United Kingdom	54 (2.10%)
Argentina	42 (1.63%)
Germany	31 (1.20%)
Spain	25 (0.97%)
China	18 (0.70%)
Brazil	13 (0.50%)
Colombia	12 (0.47%)
Italy	11 (0.43%)
Route	
oral	2946 (61.11%)
other	1875 (38.89%)
Outcomes	
hospitalization	2180 (39.25%)
other serious	2359 (42.47%)
death	699 (12.59%)
life threatening	198 (3.56%)
disability	105 (1.89%)
required intervention to Prevent Permanent Impairment/Damage	7 (0.13%)
congenital anomaly	6 (0.11%)

The percentages were calculated based on report-level counts. Other serious refers to medically significant events that do not meet predefined regulatory seriousness categories such as death, life-threatening condition, hospitalization, disability, congenital anomaly, or required intervention to prevent permanent impairment/damage.

**Figure 2:**
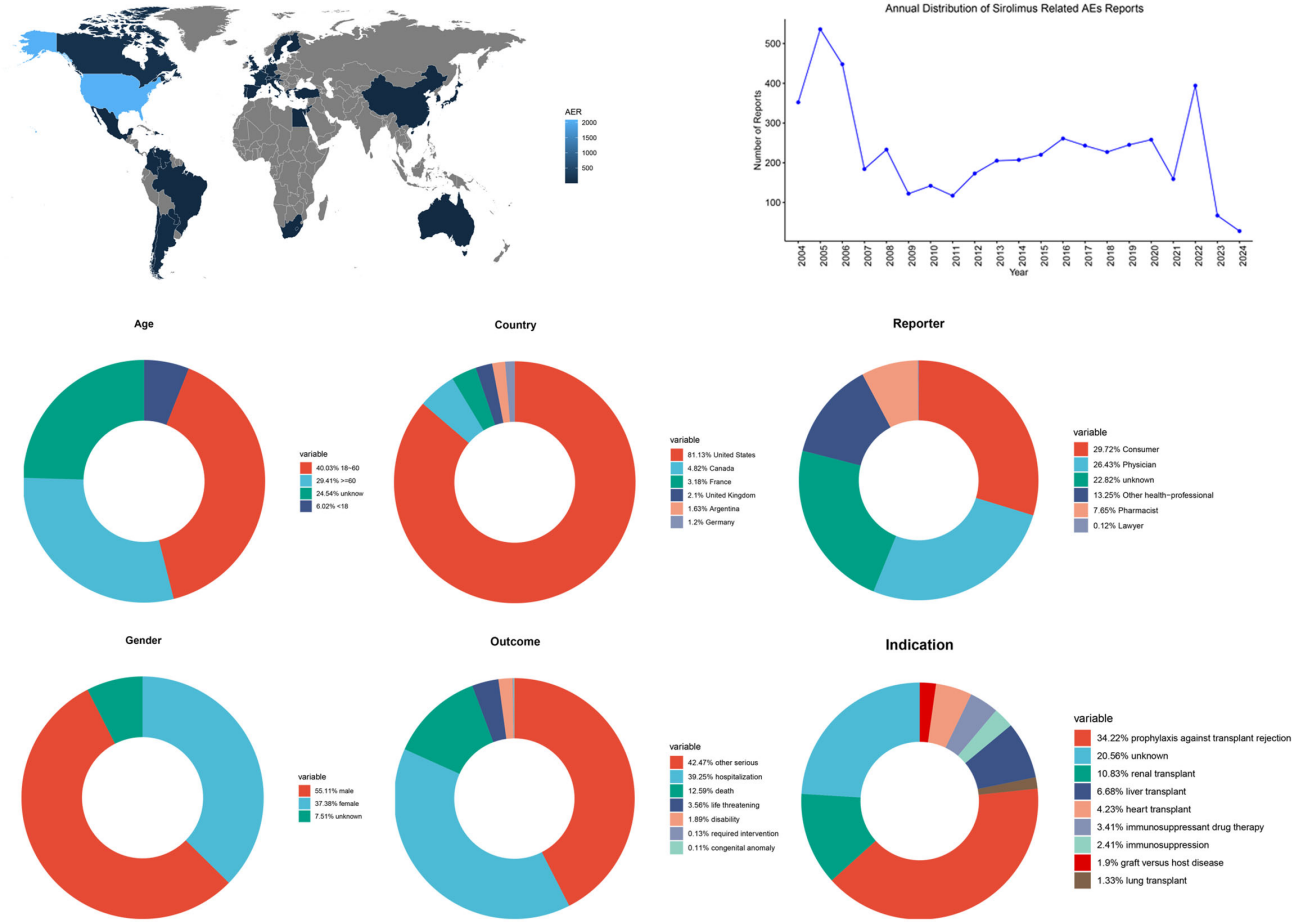
Basic characteristics of adverse events related to sirolimus from the FAERS database.

### 3.2 Signal detection for sirolimus-related ADRs based on SOC levels

**Figure 3:**
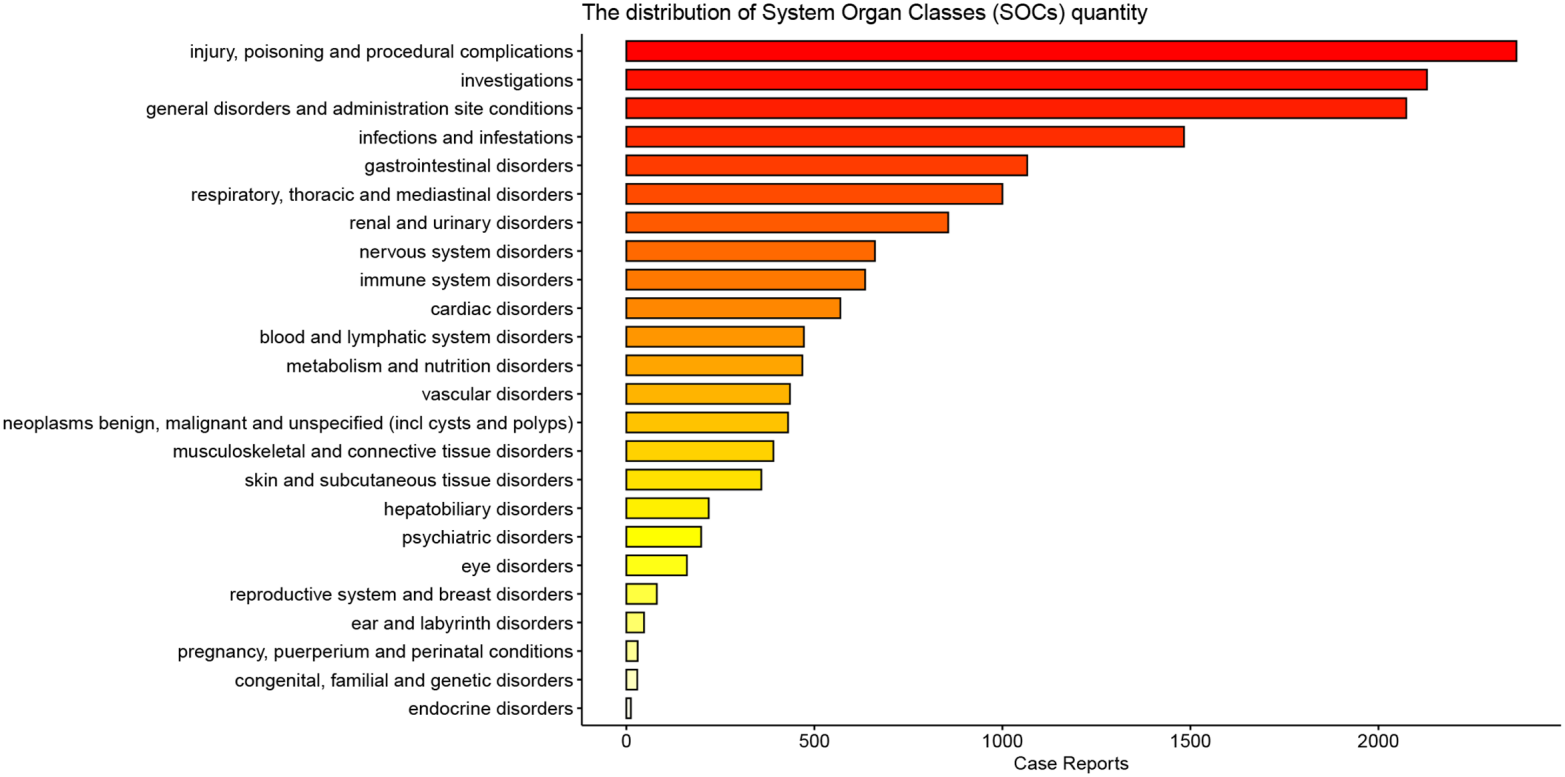
Distribution of system organ classes (SOCs) for sirolimus-related adverse events.

The analysis of various SOCs associated with sirolimus-related ADRs is shown in **Table 2**; **Figure 3**. Our statistical analysis shows that injury, poisoning and procedural complications (2,367cases) are the most common reports, followed by investigation (2,129 cases), general disorders and administration site conditions (2,074 cases), infections and infestations (1,483 cases), gastrointestinal disorders (1,066 cases), respiratory, thoracic and mediastinal disorders (1,000 cases), renal and urinary disorders (856 cases), nervous system disorders (661 cases), and immune system disorders (635 cases).

**Table 2:** Adverse drug reactions of sirolimus at the SOC level in the FAERS database (ordered by ROR).

SOC	Case reports	ROR(95% CI)	PRR (95% CI)	chisq	IC (IC025)	EBGM (EBGM05)
immune system disorders	635	3.5(3.23, 3.79)	3.4(3.14, 3.68)	1086.11	1.76(1.65)	3.4(3.18)
renal and urinary disorders	856	2.84(2.65, 3.05)	2.75(2.59, 2.92)	967.73	1.46(1.36)	2.74(2.59)
investigations	2129	2.19(2.09, 2.29)	2.03(1.95, 2.11)	1189.74	1.02(0.96)	2.03(1.95)
infections and infestations	1483	1.74(1.65, 1.84)	1.67(1.61, 1.74)	423.52	0.74(0.66)	1.67(1.6)
blood and lymphatic system disorders	472	1.66(1.52, 1.82)	1.65(1.5, 1.82)	121.49	0.72(0.59)	1.64(1.52)
injury, poisoning and procedural complications	2367	1.61(1.54, 1.68)	1.52(1.46, 1.58)	469.2	0.61(0.54)	1.52(1.47)
hepatobiliary disorders	219	1.42(1.25, 1.63)	1.42(1.24, 1.63)	27.32	0.5(0.31)	1.42(1.27)
metabolism and nutrition disorders	468	1.3(1.18, 1.42)	1.29(1.17, 1.42)	31.15	0.37(0.23)	1.29(1.19)
cardiac disorders	569	1.27(1.16, 1.38)	1.26(1.16, 1.36)	30.68	0.33(0.21)	1.26(1.17)
respiratory, thoracic and mediastinal disorders	1000	1.26(1.18, 1.34)	1.24(1.17, 1.32)	49.31	0.31(0.22)	1.24(1.18)
vascular disorders	435	1.19(1.09, 1.31)	1.19(1.08, 1.31)	13.28	0.25(0.11)	1.19(1.1)
neoplasms benign, malignant and unspecified (incl cysts and polyps)	430	0.95(0.86, 1.05)	0.95(0.86, 1.05)	1.03	-0.07(-0.21)	0.95(0.88)
gastrointestinal disorders	1066	0.72(0.68, 0.77)	0.74(0.7, 0.78)	108.6	-0.44(-0.53)	0.74(0.7)
general disorders and administration site conditions	2074	0.66(0.63, 0.69)	0.71(0.68, 0.74)	310.21	-0.5(-0.57)	0.71(0.68)
ear and labyrinth disorders	47	0.64(0.48, 0.86)	0.64(0.48, 0.86)	9.25	-0.63(-1.04)	0.64(0.51)
reproductive system and breast disorders	81	0.58(0.46, 0.72)	0.58(0.47, 0.72)	25.05	-0.79(-1.1)	0.58(0.48)
congenital, familial and genetic disorders	29	0.55(0.38, 0.8)	0.55(0.38, 0.8)	10.42	-0.85(-1.37)	0.55(0.41)
eye disorders	161	0.47(0.4, 0.55)	0.48(0.41, 0.56)	93.94	-1.07(-1.29)	0.48(0.42)
nervous system disorders	661	0.44(0.4, 0.47)	0.46(0.43, 0.5)	459.72	-1.12(-1.23)	0.46(0.43)
musculoskeletal and connective tissue disorders	391	0.42(0.38, 0.47)	0.44(0.4, 0.49)	299.09	-1.19(-1.34)	0.44(0.4)
pregnancy, puerperium and perinatal conditions	30	0.41(0.29, 0.59)	0.41(0.29, 0.58)	25.57	-1.29(-1.79)	0.41(0.3)
skin and subcutaneous tissue disorders	359	0.38(0.34, 0.42)	0.4(0.36, 0.44)	349.4	-1.33(-1.49)	0.4(0.36)
endocrine disorders	12	0.28(0.16, 0.49)	0.28(0.16, 0.49)	22.16	-1.83(-2.62)	0.28(0.17)
psychiatric disorders	199	0.2(0.17, 0.22)	0.21(0.18, 0.24)	650.12	-2.28(-2.48)	0.21(0.18)

SOC, System Organ Class; ROR, reporting odds ratio; PRR, proportional reporting ratio; Chisq, Chi-Square; IC, Information Component; EBGM, Empirical Bayesian Geometric Mean.

When we selected SOCs that met all predefined signal detection criteria (ROR, PRR, IC, and EBGM thresholds) and arranged them in descending order of case, immune system disorders (ROR = 3.5, PRR = 3.4, χ2 = 1086.11, IC = 1.76, EBGM = 3.4), renal and urinary disorders (ROR = 2.84, PRR = 2.75, χ2 = 967.73, IC = 1.46, EBGM = 2.74), investigations (ROR = 2.19, PRR = 2.03, χ2 = 1189.74, IC = 1.02, EBGM = 2.03), infections and infestations (ROR = 1.74, PRR = 1.67, χ2 = 423.52, IC = 0.74, EBGM = 1.67), blood and lymphatic system disorders (ROR = 1.66, PRR = 1.65, χ2 = 121.49, IC = 0.72, EBGM = 1.64), injury, poisoning and procedural complications (ROR = 1.61, PRR = 1.52, χ2 = 469.2, IC = 0.61, EBGM = 1.52), hepatobiliary disorders (ROR = 1.42, PRR = 1.42, χ2 = 27.32, IC = 0.5, EBGM = 1.42), metabolism and nutrition disorders (ROR = 1.3, PRR = 1.29, χ2 = 31.15, IC = 0.37, EBGM = 1.29), cardiac disorders (ROR = 1.27, PRR = 1.26, χ2 = 30.68, IC = 0.33, EBGM = 1.26), respiratory, thoracic and mediastinal disorders (ROR = 1.26, PRR = 1.24, χ2 = 49.31, IC = 0.31, EBGM = 1.24), vascular disorders (ROR = 1.19, PRR = 1.19, χ2 = 13.28, IC = 0.25, EBGM = 1.19), were found to maybe affected. These SOC-level signals should be interpreted as disproportionate reporting associations rather than confirmed causal toxicities.

### 3.3 Signal detection for sirolimus-related ADRs based on PT levels

**Table 3**; **Figures 4**, **5** offer an overview of specific PTs associated with sirolimus. Our statistical analysis shows that kidney transplant rejection (292 reports) represented one of the most frequently reported PT-level signals, followed by complications of transplanted kidney (136 cases), transplant rejection (103 cases), and immunosuppressant drug level increased (97 cases).

**Table 3:** Top clinical adverse reactions of sirolimus ranked by case reports in the FAERS database.

SOC	PT	Case reports	ROR (95% CI)	PRR (95% CI)	chisq	IC (IC025)	EBGM (EBGM05)
renal and urinary disorders	ureteric fistula	3	303.08(92.65, 991.48)	303.03 (93.49, 982.24)	823.4	8.11(6.62)	276.38(102.52)
	urinary fistula	8	248.14(120.78, 509.82)	248.02 (120.1, 512.2)	1823.75	7.84(6.86)	229.89(125.85)
	perinephric collection	8	216.06(105.52, 442.38)	215.95 (104.57, 445.97)	1601.21	7.66(6.68)	202.08(110.95)
	urinoma	5	122.35(50.06, 299.03)	122.32 (49.65, 301.34)	579	6.88(5.7)	117.75(55.75)
	glomerulonephritis proliferative	7	69.39(32.81, 146.78)	69.36 (32.93, 146.07)	461.43	6.08(5.07)	67.88(36.27)
	renal necrosis	4	63.92(23.75, 172.03)	63.9 (23.52, 173.63)	242.72	5.97(4.69)	62.65(27.36)
	renal vessel disorder	3	46.74(14.95, 146.18)	46.74 (15, 145.68)	132.3	5.53(4.1)	46.06(17.74)
investigations	immunosuppressant drug level decreased	39	120.84(87.73, 166.45)	120.55 (88.1, 164.95)	4452.53	6.86(6.4)	116.12(88.83)
	x-ray limb abnormal	3	120.46(38.02, 381.67)	120.43 (37.89, 382.78)	342.17	6.86(5.41)	116.01(44.2)
	ultrasound kidney abnormal	5	117.75(48.21, 287.6)	117.72 (47.78, 290.01)	557.66	6.83(5.65)	113.49(53.76)
	immunosuppressant drug level increased	97	114.48(93.43, 140.27)	113.8 (93.54, 138.44)	10465.71	6.78(6.49)	109.84(92.67)
	biopsy kidney abnormal	6	102.14(45.29, 230.36)	102.11 (45.72, 228.07)	581.73	6.63 (5.54)	98.91(50.09)
	culture wound positive	4	47.82(17.81, 128.38)	47.81 (17.94, 127.39)	180.55	5.56 (4.28)	47.1(20.61)
	drug level fluctuating	10	41.28(22.12, 77.05)	41.26 (22.04, 77.25)	387.69	5.35 (4.49)	40.73(24.16)
injury, poisoning and procedural complications	renal lymphocele	4	305.56(109.44, 853.18)	305.49 (110.25, 846.51)	1106.06	8.12 (6.79)	278.42(117.92)
	complications of transplanted kidney	136	233.14(195.74, 277.69)	231.19 (193.8, 275.79)	29028.41	7.75 (7.5)	215.36(186.05)
	complications of transplant surgery	19	129.77(81.99, 205.38)	129.62 (82.58, 203.45)	2328.5	6.96 (6.31)	124.5(84.79)
	vascular graft complication	4	79.29(29.39, 213.92)	79.27(29.17, 215.39)	301.51	6.27(4.99)	77.34(33.71)
	graft complication	9	54.02(27.94, 104.42)	53.99(27.73, 105.13)	460.12	5.73(4.83)	53.09(30.58)
	transplant dysfunction	24	50.37(33.65, 75.42)	50.3(33.33, 75.91)	1141.41	5.63(5.06)	49.52(35.33)
	complications of transplanted liver	5	47.75(19.74, 115.5)	47.73(19.76, 115.3)	225.33	5.56(4.39)	47.03(22.46)
immune system disorders	kidney transplant rejection	292	230.41(204.39,259.73)	226.26(201.16, 254.5)	61078.27	7.72(7.55)	211.08(190.95)
	heart transplant rejection	31	110.64(77.31, 158.35)	110.43(77.6, 157.15)	3247.2	6.74(6.23)	106.7(79.05)
	liver transplant rejection	44	90.53(67.06, 122.21)	90.29(67.29, 121.15)	3776.29	6.46(6.03)	87.78(68.29)
	pancreas transplant rejection	4	72.84(27.03, 196.31)	72.82(26.8, 197.87)	276.9	6.15(4.87)	71.19(31.05)
	chronic allograft nephropathy	15	70.17(42.05, 117.08)	70.1(42.11, 116.69)	999.4	6.1(5.38)	68.59(44.69)
	transplant rejection	103	47.8(39.33, 58.11)	47.51(39.05, 57.8)	4619.87	5.55(5.27)	46.81(39.76)
vascular disorders	lymphocele	36	189.24(135.14, 265)	188.82(135.31, 263.48)	6343.29	7.48(7)	178.14(134.4)
respiratory, thoracic and mediastinal disorders	alveolar proteinosis	7	49.61(23.51, 104.69)	49.59(23.55, 104.44)	328.08	5.61(4.6)	48.83(26.14)
neoplasms benign, malignant and unspecified (incl cysts and polyps)	ovarian adenoma	3	56.6(18.07, 177.31)	56.59(18.16, 176.38)	160.91	5.8(4.37)	55.6(21.39)

SOC, System Organ Class; PT, Preferred term; ROR, reporting odds ratio; PRR, proportional reporting ratio; Chisq, Chi-Square; IC, Information Component; EBGM, Empirical Bayesian Geometric Mean.

**Figure 4:**
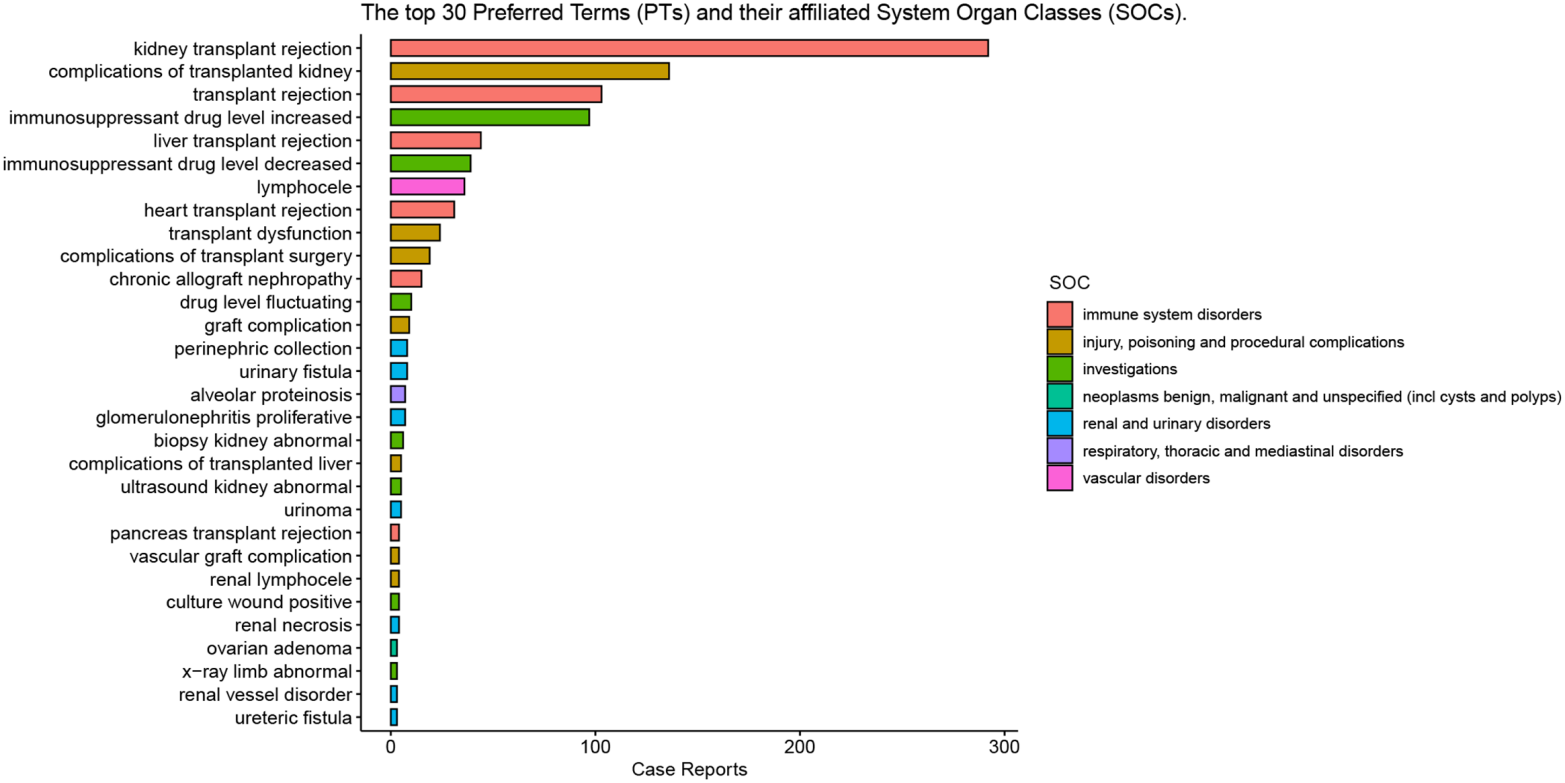
Top 30 preferred terms (PTs) of adverse events and their affiliated system organ classes (SOCs) reported for sirolimus.

**Figure 5:**
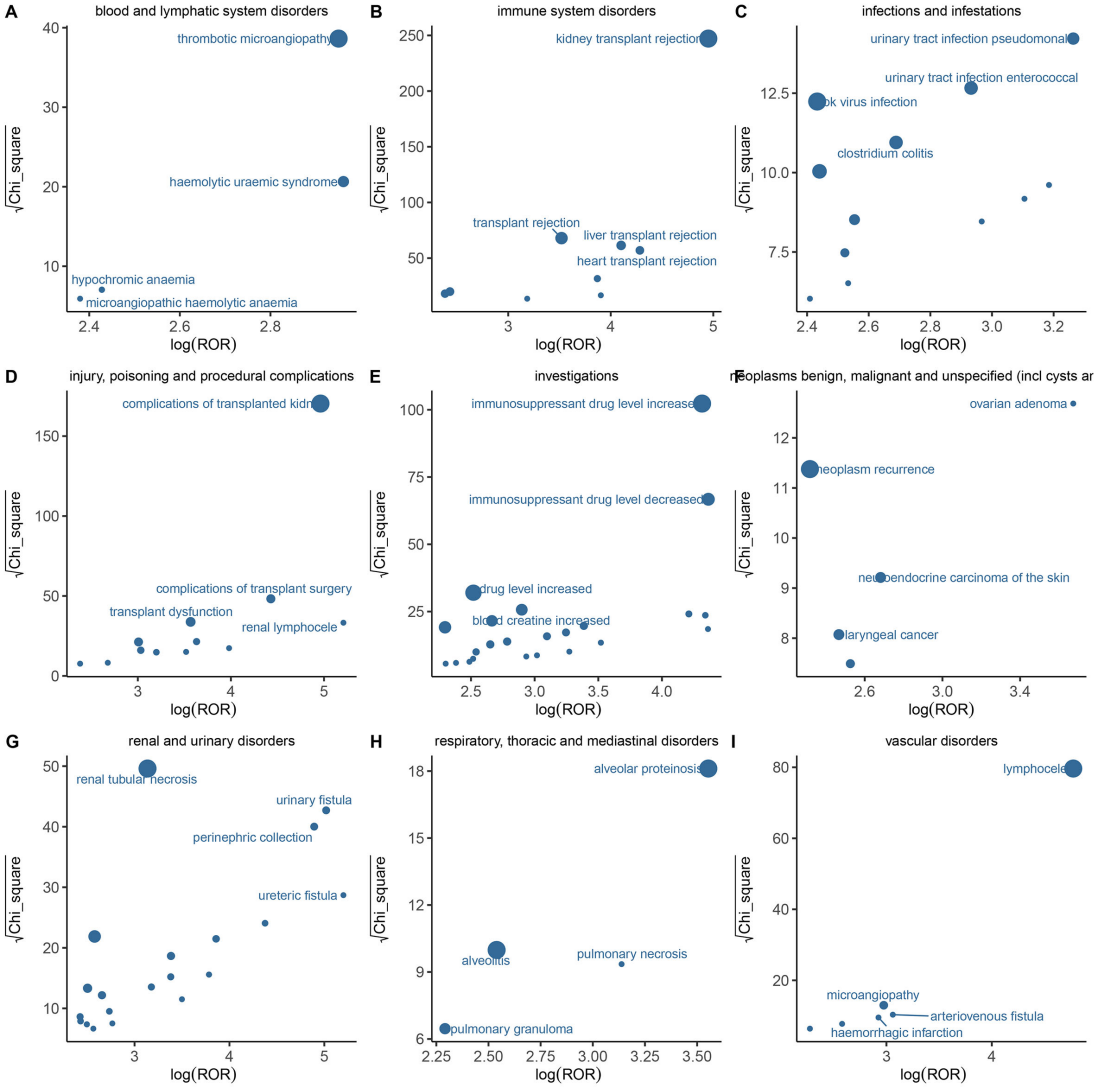
Logarithmic representation of Reporting Odds Ratios (ROR) for different adverse events linked to sirolimus.

Among these, kidney transplant rejection was notably prevalent (ROR = 230.41, PRR = 226.26, χ2 = 61078.27, IC = 7.72, EBGM = 211.08). Renal lymphocele had the highest reporting odds ratio (ROR = 305.56, PRR = 305.49, χ2 = 1106.06, IC = 8.12, EBGM = 278.42), indicating a strong disproportionality signal associated with sirolimus.

The analysis also revealed adverse reactions not prominently mentioned in the prescribing information, such as rare but notable ovarian adenoma (ROR = 56.6, PRR = 56.59, χ2= 160.91, IC = 5.8, EBGM = 55.6), which enhances the comprehensive understanding of the safety of sirolimus.

### 3.4 Subgroup analysis

Subgroup analyses revealed that males had a higher frequency of reported ADRs, particularly for kidney transplant rejection and complications of transplanted kidney (**Table 4**). Furthermore, the same trend has taken place among females (**Table 5**).

**Table 4:** Subgroup analysis of signal strength of reports of male at the preferred term level in the FAERS database.

SOC	PT	Case reports	ROR (95% CI)	PRR (95% CI)	chisq	IC (IC025)	EBGM (EBGM05)
renal and urinary disorders	perinephric collection	4	134.46(48.78, 370.65)	134.4(48.5, 372.42)	494.89	6.97(5.66)	125.65(53.79)
	ureteric stenosis	6	34.63(15.44, 77.67)	34.61(15.5, 77.3)	192.37	5.09(4.01)	34.01(17.31)
	ureteric fistula	3	383.16(110.9, 1323.77)	383.03(111.42, 1316.74)	952.59	8.32(6.76)	319.36(113.18)
	urinary fistula	5	171.09(68.52, 427.21)	171(68.07, 429.6)	775.75	7.3(6.09)	157.06(73.04)
	urinoma	3	78.73(24.81, 249.85)	78.71(24.76, 250.18)	221.07	6.24(4.79)	75.64(28.78)
	renal necrosis	3	69.25(21.88, 219.14)	69.22(21.78, 220.01)	194.68	6.06(4.62)	66.84(25.49)
	glomerulonephritis proliferative	5	55.7(22.89, 135.57)	55.67(23.04, 134.48)	260.88	5.76(4.58)	54.13(25.72)
investigations	immunosuppressant drug level decreased	21	77.97(50.38, 120.68)	77.79(50.54, 119.73)	1529.81	6.22(5.61)	74.79(51.9)
	immunosuppressant drug level increased	56	74.27(56.82, 97.07)	73.81(56.1, 97.11)	3873.32	6.15(5.77)	71.11(56.84)
	drug level fluctuating	6	34.84(15.54, 78.14)	34.82(15.59, 77.77)	193.58	5.1(4.02)	34.22(17.41)
	ultrasound kidney abnormal	3	122.29(38.05, 392.96)	122.24(37.71, 396.23)	339.11	6.85(5.38)	114.97(43.29)
	biopsy kidney abnormal	3	55.26(17.53, 174.18)	55.25(17.38, 175.61)	155.31	5.75(4.31)	53.72(20.56)
	culture wound positive	3	47.11(14.98, 148.13)	47.09(15.11, 146.77)	132.1	5.52(4.09)	45.99(17.63)
	culture stool positive	3	44.55(14.18, 139.99)	44.54(14.29, 138.82)	124.78	5.44(4.01)	43.55(16.71)
	sputum culture positive	8	38.43(19.08, 77.41)	38.4(18.96, 77.76)	285.68	5.24(4.28)	37.66(20.96)
injury, poisoning and procedural complications	complications of transplanted kidney	59	105.02(80.74, 136.59)	104.33(80.86, 134.61)	5726.75	6.63(6.25)	99(79.45)
	complications of transplant surgery	7	60.16(28.34, 127.7)	60.12(28.55, 126.61)	394.56	5.87(4.85)	58.32(31.07)
	vascular graft complication	3	70.09(22.14, 221.87)	70.07(22.05, 222.72)	197.04	6.08(4.63)	67.63(25.79)
	complications of transplanted liver	3	35.26(11.25, 110.49)	35.25(11.31, 109.87)	98.03	5.11(3.68)	34.63(13.32)
	seroma	5	31.83(13.15, 77.05)	31.81(13.17, 76.84)	146.79	4.97(3.8)	31.31(14.94)
immune system disorders	kidney transplant rejection	199	185.3(159.99, 214.61)	181.23(158, 207.88)	32588.1	7.37(7.16)	165.65(146.49)
	heart transplant rejection	14	70.48(41.33, 120.2)	70.37(41.45, 119.46)	923.52	6.09(5.34)	67.91(43.45)
	liver transplant rejection	21	55.22(35.77, 85.25)	55.09(35.79, 84.79)	1084.21	5.74(5.13)	53.58(37.26)
	chronic allograft nephropathy	8	42.83(21.25, 86.34)	42.8(21.14, 86.67)	319.43	5.39(4.43)	41.88(23.3)
	lung transplant rejection	4	28.49(10.61, 76.48)	28.48(10.69, 75.88)	104.5	4.81(3.53)	28.08(12.29)
infections and infestations	urinary tract infection pseudomonal	5	37.14(15.32, 90)	37.12(15.37, 89.67)	172.37	5.19(4.02)	36.43(17.37)
	urinary tract infection enterococcal	4	29.48(10.98, 79.15)	29.46(11.06, 78.49)	108.33	4.86(3.58)	29.03(12.7)
vascular disorders	lymphocele	23	158.28(103.41, 242.28)	157.88(102.58, 242.99)	3312.41	7.19(6.59)	145.93(102.2)
	microangiopathy	5	28.86(11.93, 69.81)	28.84(11.94, 69.67)	132.4	4.83(3.66)	28.43(13.58)
respiratory, thoracic and mediastinal disorders	pulmonary necrosis	3	35.7(11.39, 111.87)	35.69(11.45, 111.24)	99.29	5.13(3.7)	35.05(13.48)

SOC, System Organ Class; PT, Preferred term; ROR, reporting odds ratio; PRR, proportional reporting ratio; Chisq, Chi-Square; IC, Information Component; EBGM, Empirical Bayesian Geometric Mean.

**Table 5:** Subgroup analysis of signal strength of reports of female at the preferred term level in the FAERS database.

SOC	PT	Case reports	ROR (95% CI)	PRR (95% CI)	chisq	IC (IC025)	EBGM (EBGM05)
renal and urinary disorders	perinephric collection	4	330.97(119.67, 915.34)	330.77(119.37, 916.56)	1221.15	8.26(6.94)	307.21(131.15)
	renal tubular atrophy	4	61.68(22.98, 165.56)	61.65(23.14, 164.26)	235.29	5.93(4.65)	60.79(26.61)
	glomerulosclerosis	3	53.77(17.22, 167.95)	53.75(17.25, 167.53)	153.39	5.73(4.3)	53.1(20.47)
	ureteric stenosis	3	46.42(14.88, 144.86)	46.4(14.89, 144.62)	131.85	5.52(4.1)	45.92(17.72)
	renal tubular necrosis	32	38.56(27.21, 54.66)	38.38(26.97, 54.62)	1154.89	5.25(4.75)	38.05(28.42)
	kidney fibrosis	5	35.16(14.58, 84.8)	35.13(14.54, 84.86)	164.45	5.12(3.96)	34.85(16.68)
investigations	immunosuppressant drug level decreased	13	141.44(81.36, 245.87)	141.16(81.54, 244.37)	1751.7	7.09(6.33)	136.71(86.07)
	immunosuppressant drug level increased	38	162.87(117.7, 225.36)	161.94(118.35, 221.59)	5857.62	7.29(6.82)	156.1(118.95)
	drug level fluctuating	4	52.15(19.45, 139.82)	52.12(19.56, 138.87)	198.16	5.69(4.41)	51.51(22.57)
	drug level below therapeutic	17	47.62(29.51, 76.84)	47.5(29.68, 76.03)	765.41	5.55(4.88)	46.99(31.48)
	Pco_2_ decreased	4	38.16(14.26, 102.15)	38.14(14.31, 101.62)	143.38	5.24(3.97)	37.81(16.59)
injury, poisoning and procedural complications	complications of transplanted kidney	47	326.7(242.64, 439.88)	324.4(241.77, 435.27)	14090.1	8.24(7.81)	301.71(235.23)
	complications of transplant surgery	5	127.31(52.3, 309.94)	127.22(52.66, 307.33)	608.14	6.95(5.77)	123.59(58.7)
	transplant dysfunction	23	132.32(87.32, 200.5)	131.87(87.38, 199.02)	2898.28	7(6.41)	127.97(90.38)
	graft complication	6	120.11(53.34, 270.42)	120(53.73, 268.03)	688.83	6.87(5.78)	116.77(59.21)
	graft loss	3	75.03(23.96, 235.01)	75(24.06, 233.76)	215.28	6.2(4.77)	73.73(28.36)
	transplant failure	10	66.56(35.62, 124.35)	66.46(35.5, 124.44)	634.95	6.03(5.17)	65.46(38.8)
immune system disorders	kidney transplant rejection	90	279.41(225.47, 346.25)	275.64(222.18, 341.96)	23145.42	8.02(7.71)	259.09(216.53)
	heart transplant rejection	9	181.08(92.91, 352.95)	180.84(92.87, 352.13)	1544.66	7.44(6.52)	173.58(99.31)
	liver transplant rejection	15	114.41(68.48, 191.16)	114.16(68.58, 190.03)	1639.03	6.8(6.08)	111.23(72.39)
	chronic allograft nephropathy	5	90.78(37.43, 220.2)	90.72(37.55, 219.15)	434.48	6.47(5.3)	88.86(42.34)
	transplant rejection	50	82.14(62.03, 108.78)	81.53(61.97, 107.27)	3903.54	6.32(5.92)	80.03(63.27)
infections and infestations	bacterial pyelonephritis	3	79.18(25.27, 248.12)	79.14(25.39, 246.66)	227.28	6.28(4.85)	77.73(29.89)
	pneumonia streptococcal	4	37.33(13.95, 99.92)	37.31(14, 99.41)	140.13	5.21(3.94)	37(16.23)
	clostridium colitis	5	34.32(14.23, 82.76)	34.29(14.19, 82.84)	160.32	5.09(3.93)	34.03(16.29)
vascular disorders	lymphocele	12	206.77(115.8, 369.2)	206.4(114.64, 371.6)	2340.52	7.62(6.82)	196.99(121.28)
	arteriovenous fistula	3	74.17(23.68, 232.28)	74.14(23.79, 231.08)	212.79	6.19(4.76)	72.9(28.05)
	venoocclusive disease	4	35.63(13.31, 95.35)	35.61(13.36, 94.88)	133.45	5.14(3.87)	35.33(15.5)
respiratory, thoracic and mediastinal disorders	alveolar proteinosis	5	111.48(45.87, 270.97)	111.4(46.11, 269.11)	533.22	6.76(5.59)	108.61(51.66)
neoplasms benign, malignant and unspecified (incl cysts and polyps)	ovarian adenoma	3	80.66(25.73, 252.82)	80.62(25.87, 251.27)	231.57	6.31(4.88)	79.16(30.43)

SOC, System Organ Class; PT, Preferred term; ROR, reporting odds ratio; PRR, proportional reporting ratio; Chisq, Chi-Square; IC, Information Component; EBGM, Empirical Bayesian Geometric Mean.

### 3.5 Time to onset of ADRs

**Figure 6:**
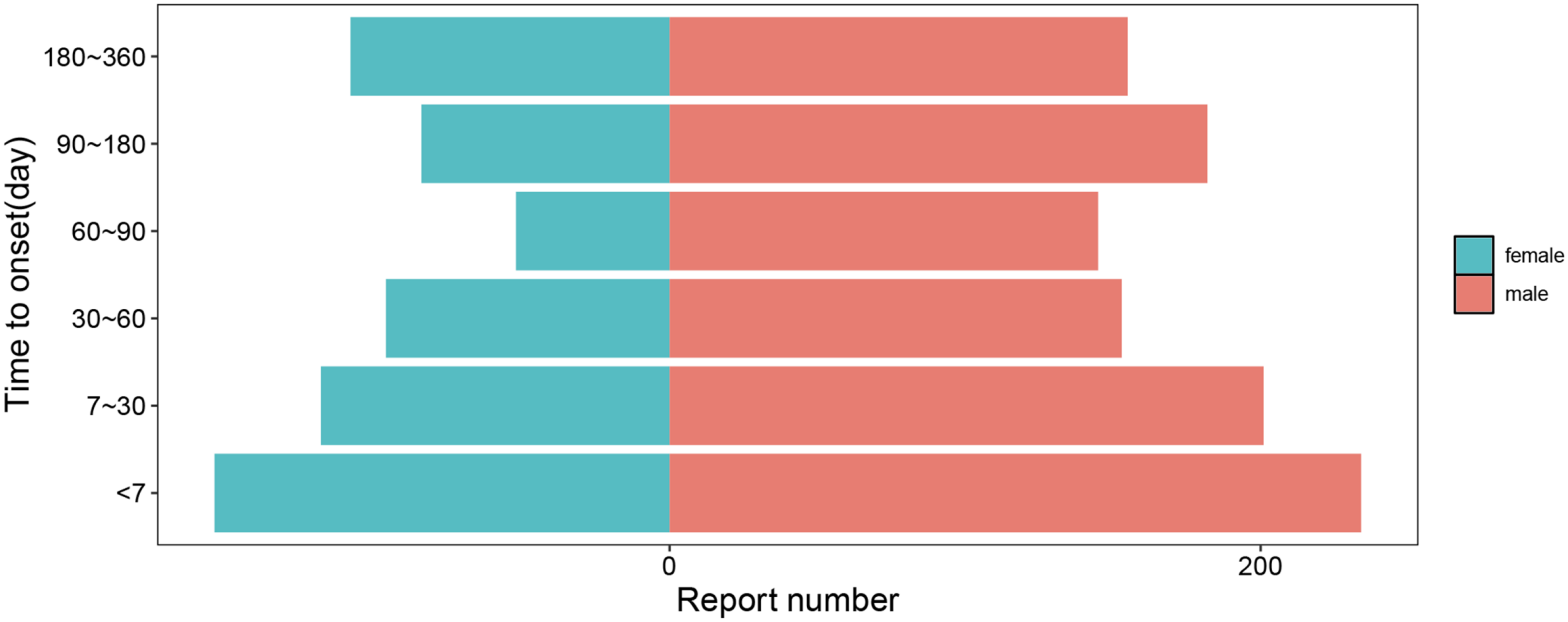
Time to onset of adverse drug reactions for sirolimus-related adverse events.

The time to onset of ADRs varied significantly among patients (**Figure 6**). Most reported adverse events occurred within the first 60 days after sirolimus initiation. Male reports were numerically more common than female reports; however, FAERS data do not permit estimation of true incidence rates.

## 4 Discussion

Sirolimus played an essential role in organ transplantation and treatment of tumors (9). Current research on sirolimus predominantly focuses on preclinical mechanisms, controlled clinical trials, and literature syntheses, with limited real-world pharmacovigilance evidence (20, 21). To our knowledge, this represents one of the few comprehensive pharmacovigilance analyses evaluating sirolimus-associated adverse event signals using the FAERS database. This pharmacovigilance approach provides critical insights to inform evidence-based risk mitigation strategies and optimize therapeutic decision-making for clinicians and regulators. Importantly, disproportionality analyses based on spontaneous reporting systems are intended for signal detection rather than causal inference. Consequently, elevated ROR or PRR values should not be interpreted as direct evidence that sirolimus caused specific adverse events. Several highly reported PTs, including renal transplant rejection and complications, may be affected by indications, disease severity, modification of immunosuppressive regimen, drug non-compliance or clinical progress related to transplantation. Therefore, these signals should be carefully interpreted in a wider context of transplantation.

Other serious medical events accounted for 42.47% of reports, followed by hospitalization (39.25%). These findings emphasize the possible threat of some ADRs related to sirolimus, underscoring the necessity for health care providers to closely follow patients and deal with serious ADRs promptly. The incidence of this serious consequence is consistent with data from other studies, which also reported significant rates of other serious and hospitalizations among sirolimus recipients (22, 23).

Disproportionality analysis identified significant ADRs associated with sirolimus across various SOCs including injury, poisoning and procedural complications, investigations, infections and infestations, respiratory, thoracic and mediastinal disorders mainly, which are consistent with previous reports (24, 25). The appearance of these symptoms emphasizes the need for doctors to closely monitor patients and manage these side effects.

At the PT level, our study showed that the ADRs with high frequency occurrence included transplant rejection, complications of transplanted kidney, and immunosuppressant drug level fluctuation, as has been reported (23, 26). Because transplant rejection-related PTs may reflect underlying disease progression, insufficient immunosuppression, treatment modification, or confounding by indication, these findings should be interpreted cautiously within the clinical transplantation context. Furthermore, being different from the reported improvement signal associated with sirolimus on neoplasms benign, malignant and unspecified (incl cysts and polyps), a signal of ovarian adenoma associated with sirolimus was identified in this study. Nevertheless, this finding may also reflect reporting artifacts, surveillance bias in transplant recipients, MedDRA coding variability, or spurious disproportionality. Therefore, this observation should be regarded as hypothesis-generating and requires further validation in prospective studies.

There are some limitations in our research. As FAERS relies on spontaneous reporting, data are subject to underreporting, selective reporting biases (e.g., overrepresentation of severe or novel events), and variable data completeness. The absence of detailed clinical records—including comorbidities, precise dosing regimens, treatment duration, concomitant medications, and surgical histories—precludes assessment of causality and confounds risk quantification for specific adverse events. These limitations may influence signal strength and interpretation. Nevertheless, our findings identify clinically significant safety signals that warrant targeted pharmacoepidemiological investigation. These results provide hypothesis-generating evidence to guide future prospective studies and enhance risk mitigation strategies for sirolimus therapy.

## 5 Conclusion

This analysis details the ADR profile of sirolimus, revealing both known and potentially novel clinically significant adverse event signals that augment its known safety profile. Ongoing pharmacovigilance and targeted investigations are necessary to confirm these pharmacovigilance signals and inform optimal safety management strategies for sirolimus.

## Data Availability

The raw data supporting the conclusions of this article will be made available by the authors, without undue reservation.
